# Synergistic effect of periodontitis and C-reactive protein levels on mortality: NHANES 2001–2004

**DOI:** 10.1371/journal.pone.0309476

**Published:** 2024-10-25

**Authors:** Miyeun Han, Whanhee Lee, Seoyeong Ahn, Moon Ho Kang, Hyeon Seok Hwang, Soie Kwon, Yaerim Kim, Jeonghwan Lee, Dong Ki Kim, Chun Soo Lim, Yon Su Kim, Jung Pyo Lee

**Affiliations:** 1 Department of Internal Medicine, National Medical Center, Seoul, Republic of Korea; 2 School of Biomedical Convergence Engineering, Pusan National University, Yangsan, Republic of Korea; 3 Department of Information Convergence Engineering, Pusan National University, Yangsan, Republic of Korea; 4 Director of Onsam Dental Clinic, Seoul, Republic of Korea; 5 Department of Oral and Maxillofacial Surgery, School of Dentistry, Seoul National University, Seoul, Republic of Korea; 6 Department of Internal Medicine, College of Medicine, Kyung Hee University Hospital, Kyung Hee University, Seoul, Republic of Korea; 7 Department of Internal Medicine, Chung-Ang University Heukseok Hospital, Seoul, Republic of Korea; 8 Department of Internal Medicine, Keimyung University School of Medicine, Daegu, Republic of Korea; 9 Department of Internal Medicine, Seoul National University Boramae Medical Center, Seoul, Republic of Korea; 10 Department of Internal Medicine, Seoul National University College of Medicine, Seoul, Republic of Korea; 11 Department of Internal Medicine, Seoul National University Hospital, Seoul, Republic of Korea; University of Pisa, ITALY

## Abstract

Periodontitis is associated with elevated C-reactive protein (CRP) levels. Although the coexistence of periodontitis and elevated CRP levels may heighten the risk of mortality, previous studies have not confirmed their synergistic effect. Understanding this interaction is crucial for identifying potential interventions to reduce mortality risk in individuals with periodontitis. This study aimed to assess the synergistic effects of periodontitis and elevated CRP levels on mortality in 7,938 adult individuals who participated in the National Health and Nutrition Examination Study 2001–2004. The association of periodontitis status and CRP levels with mortality was assessed using a survey-weighted Cox model. The interactive effect was estimated; the synergistic effect of CRP levels and periodontitis status on mortality was assessed using the relative excess risk due to interaction (RERI). Periodontitis was diagnosed in 1,065 (13.4%) participants. Compared with the participants without periodontitis and possessing CRP levels of ≤ 0.5 mg/dL, those with periodontitis (hazard ratio [HR], 1.38) or CRP levels of > 0.5 mg/dL (HR 1.23) had higher HRs. The participants with both periodontitis and CRP levels of > 0.5 mg/dL had the highest HR of 2.01. The additive scale interactive effect of the periodontal status and CRP levels, measured using RERI 0.41 (-0.07, 0.95), was positive and nearly significant in the total population. The synergy between the periodontal status and CRP levels was more prominent in the participants aged ≥60 years than that in younger individuals. Periodontitis with high CRP levels may indicate a high mortality rate, indicating the importance of active monitoring and intensive management of periodontitis and inflammatory markers.

## Introduction

Periodontitis is a chronic inflammatory disease characterized by destruction of the periodontal tissues, formation of periodontal pockets around the teeth, and gingival recession. It is a major public health concern owing to its high prevalence and negative impact on oral health. According to National Health and Nutrition Examination Survey (NHANES) 2009–2014, over 40% of adults in the United States have periodontitis [1–3]. Periodontitis causes tooth loss, pain, and functional limitations, affecting dietary choices and nutrition. Treating periodontitis and its complications incurs significant healthcare costs and productivity losses [4, 5]. Beyond oral health, periodontitis is linked to systemic conditions like cardiovascular diseases (CVD), diabetes mellitus (DM), and respiratory diseases: all exacerbated by systemic inflammation [6]. For instance, Individuals with periodontitis are at an increased risk of developing CVDs [7, 8].

CRP, an acute-phase protein produced by the liver in response to inflammation, is a biomarker for systemic inflammation. Elevated CRP levels are associated with an increased risk of various non-communicable diseases (NCDs) and mortality, particularly CVD such as coronary artery disease and stroke [9–11]. CRP contributes to atherosclerosis by promoting endothelial dysfunction, plaque formation, and rupture. In addition, elevated CRP levels are associated with insulin resistance and development of DM [12, 13].

Periodontitis induces the release of inflammatory mediators that stimulate hepatocytes to produce CRP [14]. The serum CRP levels are elevated in severe periodontitis [14–16], and periodontal therapy can reduce these levels over time [16]. Oral microbial dysbiosis may directly induce systemic inflammation through toxin release or microbial product transport into the bloodstream [17]. Recent studies have highlighted the roles of galectin-3, Nod-like receptor pyrin domain-containing protein-3 (NLRP3), and soluble urokinase-type plasminogen activator receptor (suPAR) in periodontitis progression and their interactions with CRP [18–20].

While the coexistence of periodontitis and elevated CRP levels may increase the risk of mortality; no previous study has confirmed their synergistic effect. Elevated CRP levels in patients with periodontitis indicate systemic inflammation and disease severity, contributing to endothelial dysfunction, atherosclerosis, and increased risks of cardiovascular events and mortality. Measuring CRP levels can help identify high-risk individuals, and effective treatment of periodontitis can reduce CRP levels and systemic complications. Examining the interactive roles between periodontitis and CRP levels is crucial for assessing additional damages in patients with periodontitis and suggesting effective treatment strategies. The relative excess risk due to the interaction (RERI) represents the increase in the risk from the interaction of these factors.

This study uses NHANES 2001–2004 data to elucidate the synergistic impact of periodontitis and elevated CRP levels on mortality. Understanding this interaction is vital for identifying interventions to reduce mortality risk in affected individuals.

## Methods

### Study population

The NHANES is a nationally representative survey designed to provide estimates of common chronic conditions and associated risk factors from a representative sample of the civilian, non-institutionalized population of the United States. Full descriptions about the sample design of the NHANES datasets are publicly available (https://www.cdc.gov/nchs/nhanes/). To preserve the population representativeness of the NHANES, we did not apply any inclusion or exclusion criteria during the sample population selection. Thus, adults who participated in the NHANES 2001–2004 were included in this study. More specifically, the participants with oral health data from two NHANES cohorts, 2001–2002 (n = 7130) and 2003–2004 (n = 6718) were included in this study. Nevertheless, due to the data availability, we had to exclude those under 18 years of age (n = 2,953) and those who did not complete the periodontal examination (n = 2,957). Therefore, data of a total of 7,938 participants were included in the final analysis.

### Ethics approval

The protocol for NHANES was approved by the National Center for Health Statistics (NCHS) Institutional Review Board [21] and written informed consent was obtained from all participants.

### Data collection and definition of variables

First, we collected the NHANES data (including demographic data, examination data, laboratory data, and questionnaire data) from 2001 to 2004. The NHANES data is publicly available on the official NHANES website (https://www.cdc.gov/nchs/nhanes/index.htm); we downloaded the datasets from the website. We also obtained the National Death Index (NDI) mortality data (mortality date and causes) of the adult participants included in our NHANES data (2001–2004) to examine the association between risk factors and mortality.

The demographic variables considered in this study included age, sex, ethnicity, education, and smoking status assessed using self-reported information. The body mass index (BMI) was recorded by a trained examiner at the mobile examination center. Laboratory variables, such as serum albumin and creatinine levels, were measured and analyzed in accordance with a standardized protocol (http://www.cdc.gov/nchs/nhanes.htm). The racial-ethnic groups were categorized into five categories: Mexican American, non-Hispanic white, non-Hispanic black, other Hispanic, and other races. Smoking status was derived as a binary variable; the participants were categorized as current smokers or never/ex-smokers. The NHANES data linked to the NDI were used to assess mortality. The NDI mortality data file was updated with the mortality follow-up data through December 31, 2015.

The NHANES (2001–2002, 2003–2004) data includes oral health examination components, such as periodontal pockets, recession, loss of attachment, and bleeding, derived from the partial-mouth periodontal examination (PMPE) protocol to sample teeth and sites. We downloaded this data to determine the presence of periodontitis. Patients with a minimum of ≥ 2 sites with clinical attachment loss of ≥ 3 mm and pocket depth of ≥ 4 mm were considered to have periodontitis, as described by Eke et al. [22].

Patients with a history of DM, fasting glucose levels of > 126 mg/dL, or random glucose levels of > 200 mg/dL were considered to have DM. Patients with at least two systolic BP measurements of > 140 mmHg or diastolic BP measurements of > 90 mmHg or a history of hypertension and those receiving antihypertensive medications were considered to have hypertension. Patients with an estimated glomerular filtration rate (eGFR) of < 60 mL/min/1.73 m^2^ or urine albumin creatinine ratio of > 30 mg/g were considered to have chronic kidney disease (CKD) in accordance with the guidelines [23]. CVD events were defined as a composite of congestive heart failure, coronary heart disease, angina, and a history of heart attack or stroke. The serum and urine creatinine levels were measured using the Jaffe method, with standardization to the isotope dilution mass spectrometry reference method. The urine albumin levels were measured using a solid-phase fluorescent immunoassay. The eGFR was calculated using the CKD Epidemiology Collaboration equation [24]. The CRP levels were measured via latex-enhanced nephelometry and divided according to a cut-off value of 0.5 mg/dL[25].

### Confounders/covariates

Multiple individual-level confounders and covariates provided by the NHANES were considered to address potential estimation biases from confounders and covariates. Data regarding age, sex, race/ethnicity, and educational level (<12 years and ≥12 years) of the participants were collected as demographic characteristics. Age was considered a categorical variable (18–39 years, 40–59 years, and ≥60 years) in the statistical analysis to account for the potentially nonlinear effects of age and BMI. Smoking (never or ex-/current smoker) and BMI (<18.5, 18.5–25, 25–30, and >30) were considered in the analysis to address health behaviors. Third, DM and hypertension (present or absent) were considered comorbidities. Lastly, an indicator variable for the period (2001–2002 and 2003–2004) was adjusted during the statistical analyses to address the unknown biases due to the different NHANES periods.

### Sub-populations

The total population was stratified according to the age group (people aged 18–39 year, 40–59 year, and 60 year or older), presence of DM and hypertension (present or absent, respectively), sex, and deaths due to CVDs to evaluate the differences according to the sub-populations. The number of deceased patients and their corresponding ICD-10 codes (I00 to I99) were provided by the NHANES, and were considered as deaths due to CVD.

### Statistical analysis

A three-stage analysis was performed to evaluate the effects of periodontitis status and CRP levels on mortality and the synergistic effects between these two variables. Briefly, the individual associations of periodontitis status and CRP levels with death were estimated during the first stage. The interactive effect between the periodontitis status and CRP levels was evaluated in the second stage, with two variables showing synergy during the third stage. A multivariable survey-weighted Cox proportional hazard model was used across all stages, which could appropriately address the complex-multistage probability sampling design of NHANES [19, 20]. It comprises all potential confounders, including age, sex, ethnicity, DM/hypertension, education, and smoking, which were adjusted, except for a crude model in the first stage. All analytical procedures were repeated for the sub-populations to examine whether the associations among periodontitis, CRP, and mortality were heterogeneous by certain factors possibly related to the biological or sociodemographic pathways that could partly explain associations.

More specifically, in the first stage analysis, the associations of periodontitis status (present or absent) and CRP levels (dichotomous indicator: CRP ≤ 0.5 mg/dL and CRP > 0.5 mg/dL) with mortality were evaluated using a survey-weighted Cox proportional hazard model considering the complex-multistage probability sampling design of NHANES [26, 27]. At this stage, crude and multivariate models were used to concurrently evaluate the associations with and without the effects of confounders, respectively. However, we finally used the multivariate models to determine the estimates based on the first stage analysis.

In the second stage, the interactive effect of the CRP level and periodontitis status was estimated by adding the interaction term between these two variables to the first-stage multivariate model. Hazards ratios (HRs) were calculated for each CRP level and periodontitis status using the model with the interaction term: HR_10_ for CRP levels of > 0.5 mg/dL and no periodontitis; HR_01_ for CRP levels of ≤ 0.5 and periodontitis; and HR_11_ for CRP levels of > 0.5 mg/dL and periodontitis, where CRP levels of ≤ 0.5 and no periodontitis was the reference category. This procedure allowed for calculation of relative risks (RRs) of each category compared with the baseline (no periodontitis and CRP levels of ≤ 0.5).

Lastly, the synergistic effect of the CRP level and periodontitis status on mortality was assessed using RERI [28, 29] during the last stage. RERI was calculated using HR_11_-HR_10_-HR_01_+1 as the additive interaction using HRs; RERI of > 0 indicated a positive interaction between the CRP level and periodontitis status, which also implied synergy between these two variables [28]. Monte Carlo sampling (1,000 simulated values) was performed as described in previous studies [30, 31] to compute the 95% confidence interval [CI] of RERI. The sampling was performed based on the assumption that the coefficients for the CRP level, periodontitis status, and the interaction term between these two variables and the corresponding covariance matrix from the second-stage model follow the multivariate normal distribution, because they were estimated based on the Maximum Likelihood Estimation theory. From the Monte Carlo samples of RERI_(i),_ – where i is from 1 to 1,000 (the number of simulation) – obtained through the Monte Carlo sampling, one can calculate RERI as HR_11(i)_-HR_10(i)_-HR_01(i)_+1. Then, interval estimates for RERI could be derived from the empirical distribution. We also performed sensitivity analyses to examine whether our main results were robust to the selection of confounders and CRP cutoff.

## Results

### Baseline characteristics of the study population

The mean age of the participants was 42.0 ± 18.9 years. A total of 3875 (48.8%) participants were male individuals; 644 (8.1%) participants had DM; and 2445 (30.8%) had hypertension. Periodontitis was diagnosed in 1065 (13.4%) participants. Table 1 presents the differences between the participants with and without periodontitis.

**Table 1:** Baseline characteristics of the study participants. Values ± standard deviations (for continuous variables).

	Total (N = 7938)	No periodontitis (n = 6873)	Periodontitis (n = 1065)	p
Age (years)	42.04 ± 18.89	39.97 ± 18.33	55.4 ± 16.86	<0.001
18–39, n(%)	3998 (50.4)	3800 (55.3)	198 (18.6)	
40–59, n(%)	2209 (27.8)	1825 (26.6)	384 (36.1)	
≥60, n(%)	1731 (21.8)	1248 (18.2)	483 (45.4)	
Males, n (%)	3875 (48.8)	3216 (46.8)	659 (61.9)	<0.001
Race/ethnicity, n(%)				<0.001
Mexican American	1853 (23.3)	1565 (22.8)	288(27.0)	
Other Hispanic	305 (3.8)	265 (3.9)	40 (3.8)	
Non-Hispanic white	3827 (48.2)	3407 (49.6)	420 (39.4)	
Non-Hispanic black	1648 (20.8)	1370 (19.9)	278 (26.1)	
Other races	305 (3.8)	266 (3.9)	39 (3.7)	
Education level, n (%)				<0.001
<12 years	1818 (22.9)	1374 (20.0)	444 (41.7)	
≥12 years	5108 (64.3)	4505 (65.5)	603 (56.6)	
Unknown	1012 (12.7)	994(14.5)	18 (1.7)	
Diabetes mellitus, n (%)	644 (8.1)	469 (6.8)	175 (16.4)	<0.001
Hypertension, n (%)	2445 (30.8)	1909 (27.8)	536 (50.3)	<0.001
Chronic kidney disease, n (%)	1049 (13.2)	781 (11.3)	268 (26.1)	<0.001
Body mass index (kg/m^2^), n (%)	27.92 ± 6.32	27.83 ± 6.31	28.52 ± 6.34	0.001
Smoking, n(%)				<0.001
Never or ex-smoker	5355 (67.5)	4642 (67.5)	713 (66.9)	
Current smoker	1568 (19.8)	1236 (18.0)	332 (31.2)	
Unknown	1015 (12.8)	995 (14.5)	20 (1.9)	
Laboratory findings				
eGFR (mL/min/1.73 m^2^)	97.96 ± 23.10	99.74 ± 22.70	86.42 ± 22.33	<0.001
CRP (mg/dL)	0.42 ± 0.84	0.41 ± 0.82	0.51 ± 0.92	0.001

eGFR, estimated glomerular filtration rate; CRP, C-reactive protein.

**Fig 1:**
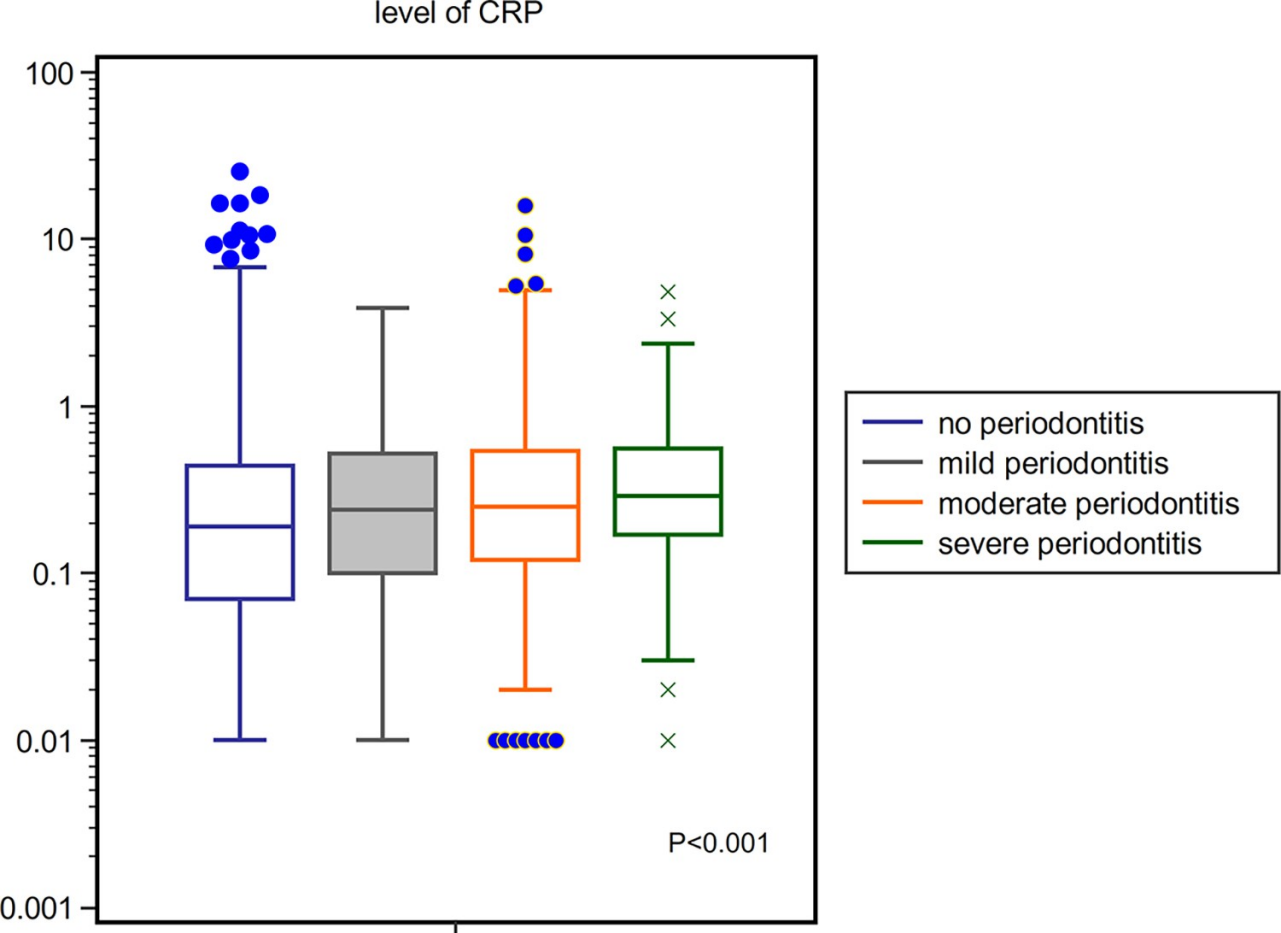
CRP levels according to the severity of periodontitis. The mild, moderate, and severe periodontitis groups comprise 276(3.5%), 700 (8.8%), 89 (1.1%) participants, respectively. The mean CRP levels in the no, mild, moderate, and severe are 0.41 ± 0.82, 0.44 ± 0.60, 0.54 ± 1.03, and 0.53 ± 0.74 mg/dL, respectively, indicating that the CRP levels gradually increased according to severity (P < 0.001). CRP, C-reactive protein.

The participants with periodontitis were older and more likely to be male patients and current smokers. These patients also had a lower educational level. Moreover, the prevalence of DM, hypertension, and CKD was higher among these patients. The eGFR was significantly lower but the CRP level was higher in the periodontitis group than those in the non-periodontitis group. For further analysis, the participants were grouped into the mild, moderate, and severe groups according to the severity of periodontitis and CRP levels. The mild, moderate, and severe groups comprised 276 (3.5%), 700 (8.8%), and 89 (1.1%) participants, respectively. The mean CRP level in the no, mild, moderate, and severe groups was 0.41 ± 0.82, 0.44 ± 0.60, 0.54 ± 1.03, and 0.53 ± 0.74 mg/dL, respectively. The CRP level was associated with the severity of periodontitis (Fig 1).

### Association of the periodontitis status and CRP levels with mortality

**Fig 2:**
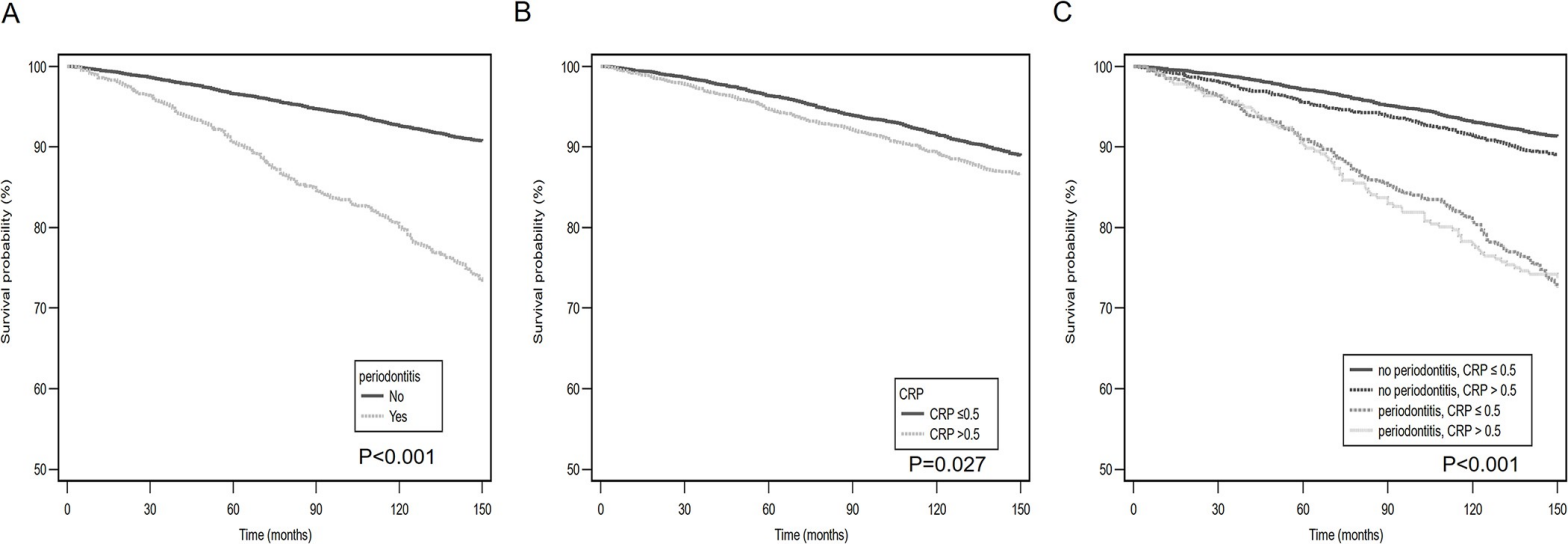
Kaplan–Meier curves for survival. The survival probability of all-cause mortality in patients (A) with and without periodontitis; (B) with CRP ≤ 0.5 mg/dL and CRP > 0.5 mg/dL; (C) without periodontitis/CRP ≤ 0.5 mg/dL, no periodontitis/CRP > 0.5 mg/dL, periodontitis/CRP ≤ 0.5 mg/dL, and periodontitis/CRP > 0.5 mg/dL. CRP, C-reactive protein.

The median follow-up duration was 12.8 years; 911 (11.5%) deaths occurred during this period. Among these 911 deaths, 199 (21.8%) were attributed to CVD. Fig 2. shows the Kaplan–Meier survival curve for mortality. Mortality was found to be higher among the participants with periodontitis (p < 0.001) and CRP levels of > 0.5 mg/dL (p = 0.027). The group without periodontitis and CRP levels of ≤ 0.5 mg/dL was found to have the best survival on dividing the groups according to the presence of periodontitis and CRP levels (Fig 2).

Crude survey-weighted Cox models (S1 Table) revealed that the periodontitis status (present or absent) and CRP levels (≤ 0.5 mg/dL or > 0.5 mg/dL) were associated with mortality, demonstrating HRs of 3.0 (95% CI, 2.54–3.55) for periodontitis status and 1.37 (95% CI, 1.08–1.74) for the CRP levels. The crude models also revealed that the relationship with mortality was evident for all confounders that were collected, except for race/ethnicity. However, the relationship with race/ethnicity became significant in the multivariate model. In addition, the associations of periodontitis status and CRP levels with mortality were also found to be significant in the multivariate model including all confounders, with HRs of 1.29 (95% CI, 1.03–1.61) and 1.44 (95% CI, 1.16–1.78) for periodontitis status and CRP level, respectively (S1 Table).

S2 Table presents the results of the CVD mortality analysis. Periodontitis was associated with CVD mortality (HR, 1.32; 95% CI, 1.14–1.54) in the multivariate model. However, no such association was observed between the CRP level and CVD mortality (HR, 1.09; 95% CI, 0.90–1.32).

### Synergistic effects of periodontitis status and CRP level on mortality

**Fig 3:**
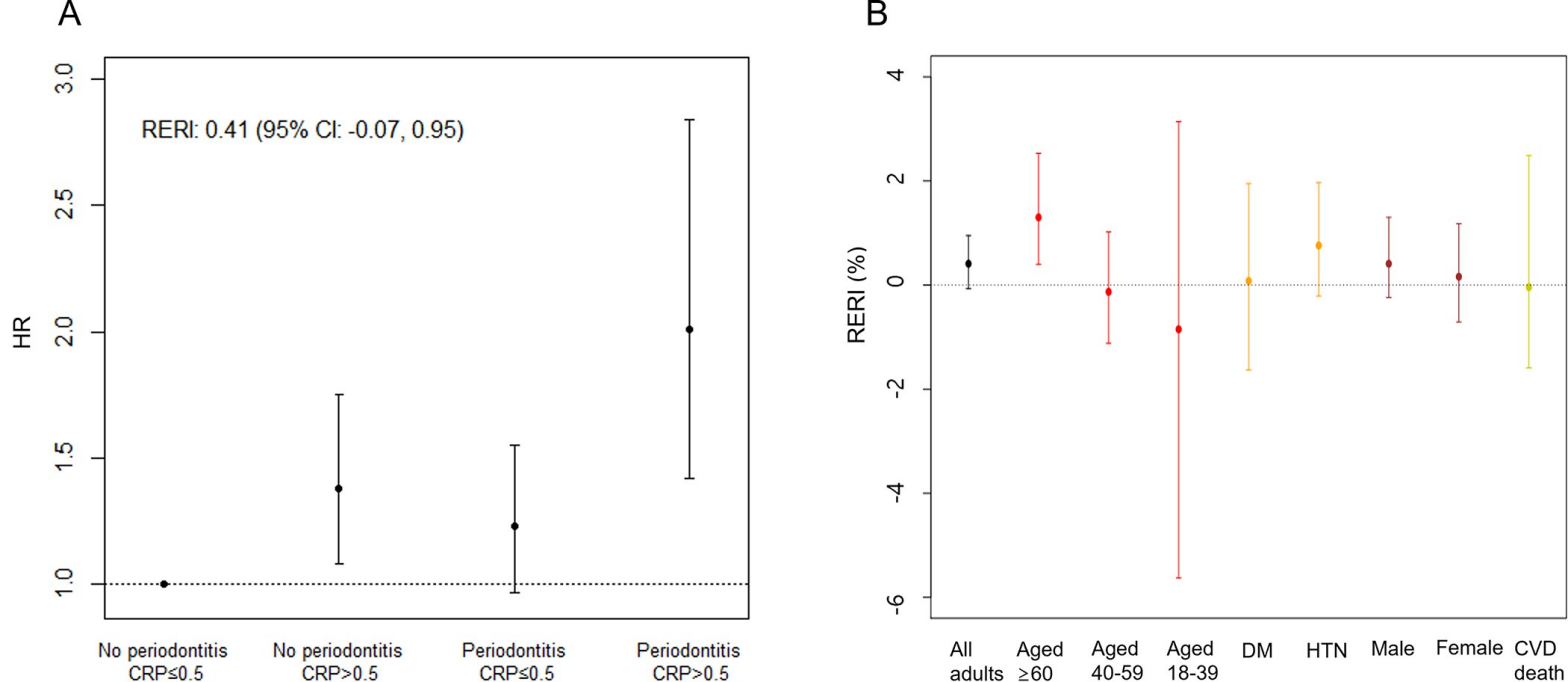
Synergic effects of periodontitis status and CRP levels on mortality. (A) The participants with both periodontitis and CRP levels of > 0.5 mg/dL have the highest HR. Moreover, the additive scale interactive effect between periodontitis and CRP status measured using RERI is marginally positive (0.41; 95% CI, -0.07 to 0.95), but the corresponding 95% CI included 0. (B) Subgroup analysis reveals that the synergy between the periodontitis status and CRP levels is more prominent in participants aged ≥ 60 years (RERI, 1.30; 95% CI, 0.40–2.53) and those with hypertension (RERI, 0.76; 95% CI, -0.21 to 1.97). CI, confidence interval; RERI, relative excess risk due to interaction; CRP, C-reactive protein.

Table 2 and Fig 3A present the association between mortality for each periodontitis status and CRP level in the total population according to the multivariate model including the interaction term between periodontitis status and CRP level. Compared with the participants without periodontitis and CRP levels of ≤ 0.5 mg/dL, the participants with periodontitis and those with CRP levels of > 0.5 mg/dL had higher HRs (1.38, 95% CI, 1.08–1.75 for periodontitis only; 1.23, 95% CI, 0.97–1.55 for CRP > 0.5 mg/dL only), and the participants with both periodontitis and CRP levels of > 0.5 mg/dL had the highest HR (2.01, 95% CI, 1.42–2.84). The additive scale interactive effect between periodontitis status and CRP levels, measured using RERI, was marginally positive (0.41, 95% CI, -0.07 to 0.95) in the total population, although the corresponding 95% CI included 0.

**Table 2:** Relative excess risk due to interaction-based assessment of the synergistic effects of C-reactive protein level and periodontitis status on mortality.

	Multivariate Cox regression		
	HR	95% CI	RERI
No periodontitis/CRP ≤ 0.5	1		
No periodontitis/CRP > 0.5	1.38	(1.08, 1.75)	
Periodontitis/CRP ≤ 0.5	1.23	(0.97, 1.55)	
Periodontitis/CRP > 0.5	2.01	(1.42, 2.84)	0.41 (-0.07, 0.95)

CI, confidence interval; RERI, relative excess risk due to interaction; CRP, C-reactive protein; HR, hazard ratio

Table 3 presents the subpopulation-specific results for the data. Based on the size of the RERI estimate, the synergy between periodontitis status and CRP levels was more prominent in the participants aged ≥ 60 years (RERI, 1.30; 95% CI, 0.40–2.53) and those with hypertension (RERI, 0.76; 95% CI, -0.21 to 1.97) than that in the total population (Fig 3B). RERI in the male participants was more pronounced than that in the female participants. However, the RERIs of both sexes and the differences were not statistically significant. Furthermore, limited evidence is available regarding the synergistic effect in the younger age groups (individuals aged 18–29 or 40–59 years), individuals with diabetes, and CVD-related deaths. S3 Table shows the race/ethnicity-specific results at baseline. The estimated RERI was pronounced in Non-Hispanic White. However, the synergism was not evident in other races/ethnicities (Non-Hispanic Black, Mexican American, other Hispanic, and other races).

**Table 3:** Subpopulation analysis of the synergistic effects of C-reactive protein level and periodontitis status on mortality in relation to age, diabetes, hypertension, sex, and deaths related to cardiovascular disease.

	HR			
	CRP = 1/Perio = 0	CRP = 0/Perio = 1	CRP = 1/Perio = 1	RERI
**Aged ≥60 years**	1.18 (0.78, 1.79)	1.27 (0.99, 1.63)	2.75 (1.81, 4.19)	1.30 (0.40, 2.53)
**Aged 40–59 years**	1.45 (0.99, 2.34)	0.78 (0.48, 1.28)	1.11 (0.49, 2.53)	-0.13 (-1.12, 1.02)
**Aged 18–39 years**	1.96 (1.20, 3.18)	2.16 (0.70, 6.65)	2.27 (0.84, 6.12)	-0.85 (-5.63, 3.14)
**Diabetes**	1.47 (0.85, 2.56)	1.20 (0.79, 1.83)	1.75 (0.90, 3.41)	0.08 (-1.63, 1.95)
**Hypertension**	1.53 (1.04, 2.25)	1.27 (1.01, 1.61)	2.57 (1.64, 4.02)	0.76 (-0.21, 1.97)
**Male**	1.54 (1.13, 2.11)	1.11 (0.81, 1.24)	2.07 (1.27, 3.37)	0.41 (-0.24, 1.30)
**Female**	1.26 (0.91, 1.74)	1.54 (1.14, 2.09)	1.96 (1.25, 3.07)	0.16 (-0.71, 1.18)
**CVD-related death**	1.64 (1.09, 2.47)	1.61 (1.09, 2.38)	2.21 (0.98, 4.99)	-0.04 (-1.59, 2.49)

CRP, C-reactive protein; Perio, periodontitis; RERI, relative excess risk due to interaction; CVD, cardiovascular disease.

Finally, the results of our sensitivity analysis were generally consistent with the main results. However, the RERI estimate size from a model with different CRP cutoff (CRP >1.0 mg/dL or not) slightly decreased (S4 Table).

## Discussion

This study showed that approximately 13% of adults had periodontitis, with an analysis of all participants revealing a positive association between CRP level and periodontitis severity. Over a median follow-up of 12.8 years, 11.5% of participants had died, with 21.8% of the mortality attributed to CVD. Those with periodontitis or high CRP level had a higher risk of death, while the presence of periodontitis and high CRP level together increased the risk of death, particularly in older individuals and those with high blood pressure. These findings suggest the importance of monitoring periodontal health and inflammation levels to help prevent the exacerbation of underlying health concerns.

Compared with those of other previously mentioned studies [1], the prevalence of periodontitis in this study appeared to be lower. First, this difference may stem from variations in diagnostic methods. The utilization of PMPE protocols in this study might have resulted in an underestimation of periodontitis cases by more than 50% [32, 33]. Second, there seems to be a gradual rise in the prevalence of periodontitis in recent years [34, 35]. Therefore, the prevalence of periodontal disease in this paper, which was based on data from 2001–2004, is lower than that of recent studies utilizing more current data.

When analyzing this study population, we used a CRP cut-off value of 0.5 mg/dL, based on reference values used in previous studies [16, 36, 37]. CRP levels between 0.0 and 0.5 mg/dL indicate normal values, whereas levels over 0.5 mg/dL are considered indicative of mild or severe inflammation. The findings of this study suggest that participants with periodontitis had elevated CRP levels and that periodontitis with CRP levels of > 0.5 mg/dL was a risk factor for mortality. Compared with the participants without periodontitis and CRP levels of ≤ 0.5 mg/dL, those with both periodontitis and CRP levels of > 0.5 mg/dL demonstrated the highest HR of 2.01. The additive scale interactive effect between periodontitis status and CRP levels was positive and nearly significant in the total population. In the subpopulation analysis, the synergy between periodontitis status and CRP levels was more prominent in individuals aged ≥60 years.

The results of the present study demonstrated that periodontitis was associated with increased CVD-related and all-cause mortality, which is consistent with the results of previous studies. Periodontal infections may also cause bacteremia, triggering host systemic inflammatory responses and chronic inflammation related to the pathogenesis of atherosclerosis [38]. Additionally, it has been reported to increase the risk of mortality due to CVDs, such as stroke [39], ischemic heart disease [40], and myocardial infarction [41]. Systemic inflammation in patients with periodontitis can potentially accelerate endothelial dysfunction, plaque build-up, and coronary heart disease events [2]. However, the association between CRP and CVD mortality was not significant in this analysis, and the primary reason for this is speculated to be the low incidence of CVD mortality due to the small sample size.

In addition to revealing an association between periodontitis and mortality, the present study confirmed the synergistic effect of periodontitis status and CRP levels on mortality. The synergy between periodontitis and CRP levels may have a biological basis, given that they both possess similar pathophysiological pathways that can result in CVD-related death or all-cause mortality. Inflammatory cells are recruited to the site of infection in patients with periodontitis, leading to the formation of reactive oxygen species. The oxidative stress leads to tissue destruction and systemic inflammation in patients with periodontitis [2]. Furthermore, CRP is an acute-phase inflammatory protein; thus, its expression is elevated under inflammatory conditions. In turn, CRP mediates inflammation and responses to infection, such as the complement pathway, apoptosis, phagocytosis, nitric oxide release, and cytokine production [42]. An elevation in the CRP levels is also a strong independent predictor of cardiovascular disease in asymptomatic individuals and they have been linked to prognosis in patients with atherosclerotic disease, suggesting that they play an active role in the pathophysiology of CVD.

Another mechanism underlying periodontitis involves the influence of systemic inflammation on periodontal tissues. Systemic inflammatory diseases, such as diabetes, hematological diseases, and viral infections, can increase the susceptibility to periodontal inflammation [43]. For instance, impairment in glucose tolerance is associated with a significant increase in the interleukin-6 and CRP levels, and the prevalence of periodontal disease in patients with DM was found to be higher than that of healthy controls [44]. Furthermore, drugs or diseases resulting in the suppression of normal inflammatory and immune mechanisms, such as HIV infection, may predispose the individual to periodontal destruction [45].

Recent studies regarding Galectin-3, NLRP3, and suPAR have provided clues on the synergistic effect in periodontitis evolution and inflammation. Galectin-3, a carbohydrate-binding protein, is known to affect the key processes involved in the pathogenesis of periodontal diseases, inflammatory/immune responses, and antimicrobial activity [46]. A recent study has shown that patients with periodontitis had higher serum and salivary Galectin-3 levels, which are associated with endothelial dysfunction and CVD risk, than did healthy individuals [19]. NLRP3, a key component of the inflammasome, contributes to the chronic inflammatory facet of periodontitis by mediating immune cell recruitment, cytokine production, and tissue destruction [47]. Additionally, its interaction with CRP underscores its significance in periodontitis pathogenesis [18]. suPAR also plays an essential function in leukocyte and endothelial homeostasis and, consequently, in the development of CVD and periodontitis [48]. Understanding the intricate interplay between Galectin-3, NLRP3, suPAR, CRP, and periodontitis requires further studies, which could lead to pivotal findings for elucidating disease mechanisms and identifying potential therapeutic targets.

This study has some limitations. First, the interval between the measurement of variables, including CRP levels, and outcomes, such as mortality, was more than 10 years, necessitating the assumption that the CRP levels measured once would be maintained at the same level for the next decade. Therefore, further validation in another study population is required. Second, periodontitis was diagnosed using the PMPE protocol, which has been reported to underestimate the true prevalence of periodontitis by ≥ 50% [32]. Nevertheless, periodontitis has been diagnosed using PMPE in previous studies [49, 50]. Third, the number of study participants may have been insufficient to confirm the synergistic effect, and the number of CVD events was not sufficient to confirm the statistical significance of synergy. However, this study is the first to demonstrate the synergy between periodontitis and CRP levels, even after adjusting for confounders. Fourth, the NHANES data we used in this study are a cross-sectional survey to assess the general health and nutritional status of people living in the United States. Thus, we were limited in examining the concomitant medications, underlying medical conditions, and/or periodontitis treatments that could be major confounders between periodontitis and CRP levels. This limitation should be addressed carefully in the future with improved datasets. Fifth, although we recognized that there was more recent NHANES data (2009–2014) that addressed periodontitis more accurately, we could not use the relatively recent data due to the sample size of the mortality. Specifically, we collected the latest NDI mortality data linked to the NHANES. However, the number of death cases was not sufficient to perform our statistical analyses (the Cox proportional hazard and the synergism models). Thus, we carefully determined to use the current NHANES data (2001–2004) that included sufficient mortality cases to examine our study objectives. Finally, this study could address the interactive roles between periodontitis and CRP at a baseline point because the NHANES was not a panel study and did not include time-varying information on periodontitis and CRP statuses. Therefore, this indicated that this study was not able to address the latent mediating roles of CRP on the relationship between periodontitis and mortality, and also implied that our study results should be interpreted carefully as the marginal interaction between periodontitis and CRP at a baseline point and the potential collider bias have to be studied with longitudinal data in future studies.

In conclusion, individuals with periodontitis and high CRP levels might show a high mortality rate. Therefore, active monitoring and intensive management of periodontitis and inflammatory markers are warranted in this population.

## Supporting information

S1 Table Association of periodontitis and C-reactive protein status with mortality according to the survey-weighted Cox proportional hazard models. (DOCX)

S2 Table Association of periodontitis and CRP status with cardiovascular mortality. (DOCX)

S3 Table Race/ethnicity-specific excess risk due to interaction-based assessment of the synergistic effects of C-reactive protein level and periodontitis status on mortality. (DOCX)

S4 Table Sensitivity analyses: Additional covariate adjustments and a different CRP cutoff. (DOCX)
