# Supplementary material for: Synergistic effect of periodontitis and C-reactive protein levels on mortality: NHANES 2001–2004
Source: PLoS One. 2024 Oct 25;19(10):e0309476. doi: 10.1371/journal.pone.0309476 (PMC11508168; doi:10.1371/journal.pone.0309476)
Supplement: S1 Table — (DOCX) [file pone.0309476.s001.docx]

**S1 Table**. Association of periodontitis and C-reactive protein status with mortality according to the survey-weighted Cox proportional hazard models

|  | **Crude (univariate) model** | | |  | **Multivariate model** | | |
| --- | --- | --- | --- | --- | --- | --- | --- |
|  | **HR** | **95% CI** | **P** |  | **HR** | **95% CI** | **P** |
| **Periodontitis** |  |  |  |  |  |  |  |
| No | 1 |  |  |  | 1 |  |  |
| Yes | 3.00 | (2.54, 3.55) | <0.001 |  | 1.29 | (1.03, 1.61) | 0.027 |
| **Ln_CRP** |  |  |  |  |  |  |  |
| CRP ≤ 0.5 | 1 |  |  |  | 1 |  |  |
| CRP > 0.5 | 1.37 | (1.08, 1.74) | 0.011 |  | 1.44 | (1.16, 1.78) | 0.002 |
| **Age** |  |  |  |  |  |  |  |
| 18-39 yr | 1 |  |  |  | 1 |  |  |
| 40-59 yr | 1.54 | (0.41, 5.80) | 0.51 |  | 2.35 | (1.68, 3.28) | <0.001 |
| ≥60 yr | 88.53 | (40.94, 191.43) | <0.001 |  | 13.15 | (9.35, 18.49) | <0.001 |
| **Sex** |  |  |  |  |  |  |  |
| Male | 1 |  |  |  | 1 |  |  |
| Female | 0.69 | (0.60, 0.79) | <0.001 |  | 0.60 | (0.52, 0.69) | <0.001 |
| **Race/Ethnicity** |  |  |  |  |  |  |  |
| Mexican American | 1 |  |  |  | 1 |  |  |
| Other Hispanic | 1.09 | (0.64, 1.86) | 0.75 |  | 1.00 | (0.62, 1.62) | 0.991 |
| Non-Hispanic White | 0.99 | (0.74, 1.34) | 0.96 |  | 0.76 | (0.59, 0.98) | 0.033 |
| Non-Hispanic Black | 1.02 | (0.74, 1.41) | 0.90 |  | 0.74 | (0.56, 0.99) | 0.039 |
| Other | 0.72 | (0.31, 1.67) | 0.43 |  | 0.65 | (0.28, 1.50) | 0.300 |
| **Education level** |  |  |  |  |  |  |  |
| <12 years | 1 |  |  |  | 1 |  |  |
| ≥12 years | 0.47 | (0.38, 0.58) | <0.001 |  | 0.64 | (0.51, 0.80) | <0.001 |
| **Diabetes mellitus** |  |  |  |  |  |  |  |
| No | 1 |  |  |  | 1 |  |  |
| Yes | 4.08 | (3.44, 4.83) | <0.001 |  | 1.69 | (1.41, 2.04) | <0.001 |
| **Hypertension** |  |  |  |  |  |  |  |
| No | 1 |  |  |  | 1 |  |  |
| Yes | 3.91 | (3.23, 4.73) | <0.001 |  | 1.67 | (1.31, 2.12) | <0.001 |
| **Body mass index** |  |  |  |  |  |  |  |
| 18.5 or less | 1 |  |  |  | 1 |  |  |
| 18.5-25 | 0.48 | (0.33, 0.70) | <0.001 |  | 0.48 | (0.32, 0.71) | <0.001 |
| 25-30 | 0.59 | (0.45, 0.79) | <0.001 |  | 0.38 | (0.30, 0.49) | <0.001 |
| 30 or over | 0.60 | (0.41, 0.87) | 0.008 |  | 0.39 | (0.28, 0.56) | <0.001 |
| **Smoking** |  |  |  |  |  |  |  |
| Never or ex-smoker | 1 |  |  |  | 1 |  |  |
| Current smoker | 1.72 | (1.44, 2.05) | <0.001 |  | 1.50 | (1.24, 1.81) | <0.001 |

HR, hazard ratio; CI, confidence interval; CRP, C-reactive protein
