# Supplementary material for: Synergistic effect of periodontitis and C-reactive protein levels on mortality: NHANES 2001–2004
Source: PLoS One. 2024 Oct 25;19(10):e0309476. doi: 10.1371/journal.pone.0309476 (PMC11508168; doi:10.1371/journal.pone.0309476)
Supplement: S2 Table — (DOCX) [file pone.0309476.s002.docx]

**S2 Table.** Association of periodontitis and CRP status with cardiovascular mortality

|  | **Crude (Univariate) model** | | |  | **Multivariate model** | | |
| --- | --- | --- | --- | --- | --- | --- | --- |
|  | **HR** | **95% CI** | **P** |  | **HR** | **95% CI** | **P** |
| **Periodontitis** |  |  |  |  |  |  |  |
| No | 1 |  |  |  | 1 |  |  |
| Yes | 2.09 | (1.81, 2.42) | <0.001 |  | 1.32 | (1.14, 1.54) | 0.002 |
| **Ln_CRP** |  |  |  |  |  |  |  |
| CRP <= 0.5 | 1 |  |  |  | 1 |  |  |
| CRP >0.5 | 1.34 | (1.13, 1.61) | 0.002 |  | 1.09 | (0.90, 1.32) | 0.400 |

HR, hazard ratio; CI, confidence interval; CRP, C-reactive protein
