# Supplementary material for: Synergistic effect of periodontitis and C-reactive protein levels on mortality: NHANES 2001–2004
Source: PLoS One. 2024 Oct 25;19(10):e0309476. doi: 10.1371/journal.pone.0309476 (PMC11508168; doi:10.1371/journal.pone.0309476)
Supplement: S3 Table — (DOCX) [file pone.0309476.s003.docx]

**S3 Table.** Race/Ethnicity-specific excess risk due to interaction-based assessment of the synergistic effects of C-reactive protein level and periodontitis status on mortality

|  | **HR** | | |  |
| --- | --- | --- | --- | --- |
|  | **CRP = 1/Perio = 0** | **CRP = 0/Perio = 1** | **CRP = 1/Perio = 1** | **RERI** |
| **Non-Hispanic White** | 1.33 (1,1.78) | 1.23 (0.93,1.62) | 2.47 (0.92,6.64) | 0.91 (0.17,3.09) |
| **Non-Hispanic Black** | 1.27 (0.82,1.95) | 1.14 (0.81,1.59) | 1.08 (0.57,2.04) | -0.33 (-1.18,0.6) |
| **Mexican American, Other Hispanic, and Other races** | 1.17 (0.68,2.01) | 1.22 (0.61,2.41) | 1.85 (0.94,3.64) | 0.47 (-0.78,1.73) |

HR, hazard ratio; CRP, C-reactive protein; RERI, relative excess risk due to interaction
