# Supplementary material for: Synergistic effect of periodontitis and C-reactive protein levels on mortality: NHANES 2001–2004
Source: PLoS One. 2024 Oct 25;19(10):e0309476. doi: 10.1371/journal.pone.0309476 (PMC11508168; doi:10.1371/journal.pone.0309476)
Supplement: S4 Table — (DOCX) [file pone.0309476.s004.docx]

**S4 Table. Sensitivity analyses: additional covariate adjustments and a different CRP cutoff**

|  | **HR** | | |  |
| --- | --- | --- | --- | --- |
|  | **CRP = 1/Perio = 0** | **CRP = 0/Perio = 1** | **CRP = 1/Perio = 1** | **RERI** |
| **Main Model** | 1.38 (1.08, 1.75) | 1.23 (0.97, 1.55) | 2.01 (1.42, 2.84) | 0.41 (-0.07, 0.95) |
| **eGFR adjusted** | 1.37 (1.07,1.76) | 1.21 (0.95,1.53) | 2.07 (1.47,2.91) | 0.49 (-0.01,1.11) |
| **SBP adjusted** | 1.39 (1.09,1.76) | 1.21 (0.95,1.54) | 1.97 (1.4,2.77) | 0.38 (-0.11,0.98) |
| **CRP >1.0** | 1.35 (1.01,1.8) | 1.27 (1.01,1.59) | 1.94 (1.25,3.01) | 0.33 (-0.46,1.23) |

HR, hazard ratio; CRP, C-reactive protein; RERI, relative excess risk due to interaction; eGFR, estimated glomerular filtration rate
